# Effect of solution saturation state and temperature on diopside dissolution

**DOI:** 10.1186/1467-4866-8-3

**Published:** 2007-03-26

**Authors:** Suvasis Dixit, Susan A Carroll

**Affiliations:** 1 Atmospheric, Earth, and Energy Department, Lawrence Livermore National Laboratory, Livermore, CA 94550, USA

## Abstract

Steady-state dissolution rates of diopside are measured as a function of solution saturation state using a titanium flow-through reactor at pH 7.5 and temperature ranging from 125 to 175°C. Diopside dissolved stoichiometrically under all experimental conditions and rates were not dependent on sample history. At each temperature, rates continuously decreased by two orders of magnitude as equilibrium was approached and did not exhibit a dissolution plateau of constant rates at high degrees of undersaturation. The variation of diopside dissolution rates with solution saturation can be described equally well with a ion exchange model based on transition state theory or pit nucleation model based on crystal growth/dissolution theory from 125 to 175°C. At 175°C, both models over predict dissolution rates by two orders of magnitude indicating that a secondary phase precipitated in the experiments.

The ion exchange model assumes the formation of a Si-rich, Mg-deficient precursor complex. Lack of dependence of rates on steady-state aqueous calcium concentration supports the formation of such a complex, which is formed by exchange of protons for magnesium ions at the surface. Fit to the experimental data yields

Rate (moldiopsidecm−2s−1)=k×10−Ea/2.303RT(aH+2aMg2+)n
 MathType@MTEF@5@5@+=feaafiart1ev1aaatCvAUfKttLearuWrP9MDH5MBPbIqV92AaeXatLxBI9gBaebbnrfifHhDYfgasaacH8akY=wiFfYdH8Gipec8Eeeu0xXdbba9frFj0=OqFfea0dXdd9vqai=hGuQ8kuc9pgc9s8qqaq=dirpe0xb9q8qiLsFr0=vr0=vr0dc8meaabaqaciaacaGaaeqabaqabeGadaaakeaaieaacqWFsbGucqWFHbqycqWF0baDcqWFLbqzcqqGGaaicqGGOaakcqWFTbqBcqWFVbWBcqWFSbaBcqWFGaaicqWFKbazcqWFPbqAcqWFVbWBcqWFWbaCcqWFZbWCcqWFPbqAcqWFKbazcqWFLbqzcqWFGaaicqWFJbWycqWFTbqBdaahaaWcbeqaaiabgkHiTiabikdaYaaakiab=bcaGiab=nhaZnaaCaaaleqabaGaeyOeI0IaeGymaedaaOGaeiykaKIaeyypa0Jaem4AaSMaey41aqRaeeymaeJaeeimaaZaaWbaaSqabeaacqGHsislcqWGfbqrdaWgaaadbaGaemyyaegabeaaliabc+caViabikdaYiabc6caUiabiodaZiabicdaWiabiodaZiabdkfasjabdsfaubaakmaabmaabaWaaSaaaeaacqWFHbqydaqhaaWcbaGaemisaG0aaWbaaWqabeaacqGHRaWkaaaaleaacqaIYaGmaaaakeaacqWFHbqydaWgaaWcbaGaemyta0Kaem4zaC2aaWbaaWqabeaacqaIYaGmcqGHRaWkaaaaleqaaaaaaOGaayjkaiaawMcaamaaCaaaleqabaGaemOBa4gaaaaa@6D9A@

where the Mg-H exchange coefficient, *n *= 1.39, the apparent activation energy, *E*_*a *_= 332 kJ mol^-1^, and the apparent rate constant, *k *= 10^41.2 ^mol diopside cm^-2 ^s^-1^.

Fits to the data with the pit nucleation model suggest that diopside dissolution proceeds through retreat of steps developed by nucleation of pits created homogeneously at the mineral surface or at defect sites, where homogeneous nucleation occurs at lower degrees of saturation than defect-assisted nucleation. Rate expressions for each mechanism (*i*) were fit to

Ri=cbiexp⁡(−Eb,ikT)KT,eqexp⁡(παT,i2ωh3(kT)2|1ln⁡Ω|)
 MathType@MTEF@5@5@+=feaafiart1ev1aaatCvAUfKttLearuWrP9MDH5MBPbIqV92AaeXatLxBI9gBaebbnrfifHhDYfgasaacH8akY=wiFfYdH8Gipec8Eeeu0xXdbba9frFj0=OqFfea0dXdd9vqai=hGuQ8kuc9pgc9s8qqaq=dirpe0xb9q8qiLsFr0=vr0=vr0dc8meaabaqaciaacaGaaeqabaqabeGadaaakeaaieaacqWFsbGudaWgaaWcbaGae8xAaKgabeaakiabg2da9iab=ngaJjab=jgaInaaBaaaleaacqWFPbqAaeqaaOGagiyzauMaeiiEaGNaeiiCaa3aaeWaaeaadaWcaaqaaiabgkHiTiab=veafnaaBaaaleaacqWFIbGycqGGSaalcqWFPbqAaeqaaaGcbaGae83AaSMae8hvaqfaaaGaayjkaiaawMcaaiab=TealnaaBaaaleaacqWFubavcqGGSaalcqWFLbqzcqWFXbqCaeqaaOGagiyzauMaeiiEaGNaeiiCaa3aaeWaaeaadaWcaaqaaGGaciab+b8aWjab+f7aHnaaDaaaleaacqWFubavcqGGSaalcqWFPbqAaeaacqaIYaGmaaGccqGFjpWDcqWGObaAaeaacqaIZaWmcqGGOaakieqacqqFRbWAcqWFubavcqGGPaqkdaahaaWcbeqaaiabikdaYaaaaaGcdaabdaqaamaalaaabaGaeGymaedabaGagiiBaWMaeiOBa4MaeuyQdCfaaaGaay5bSlaawIa7aaGaayjkaiaawMcaaaaa@6858@

where the step edge energy (α) for homogeneously nucleated pits were higher (275 to 65 mJ m^-2^) than the pits nucleated at defects (39 to 65 mJ m^-2^) and the activation energy associated with the temperature dependence of site density and the kinetic coefficient for homogeneously nucleated pits (E_b-homogeneous _= 2.59 × 10^-16 ^mJ K^-1^) were lower than the pits nucleated at defects (E_b-defect assisted _= 8.44 × 10^-16 ^mJ K^-1^).

## 1. Background

Chemical weathering of minerals play an important control on a variety of process in the Earth's near surface environment. As a consequence, a large number of studies have been devoted to quantifying dissolution rate of minerals both in the laboratory and in the field. Laboratory studies have been conducted to understand the mechanism of dissolution and also to quantify the effect of various physico-chemical conditions on dissolution rates. Despite these efforts in the last two decades, dissolution rates predicted from laboratory studies are two to several orders of magnitude higher than those measured in the field [1]. One of the causes of this discrepancy is attributed to the fact dissolution rates measured in the laboratory are mostly obtained at far-from equilibrium conditions and are extrapolated to close to equilibrium field conditions assuming a simple function of dissolution rate with respect to solution saturation. However the few studies that have been conducted in the last decade show a much more complex relation between dissolution rate and Gibbs free energy (ΔG_r_) [2-20]. The macroscopic rates have either been fit with a complex functional dependence on ΔG_r _[2-5] or fit with inferred dissolution mechanisms; such as the ion exchange model [18] or pit nucleation model [6,7].

The objectives of this study are to investigate the effect of solution saturation state and temperature on diopside dissolution and in the process develop a database against which some of the mechanistic dissolution models can be evaluated. We chose to study diopside, (CaMgSi_2_O_6_), a clinopyroxene mineral, because of its widespread occurrence in nature and also because Ca and Mg containing minerals have been targeted for geological sequestration of CO_2_. In this study we measured steady-state dissolution rates of diopside as a function of solution saturation state using a titanium flow-through reactor at pH 7.5 and temperature ranging from 125 to 175°C. Additionally, we tested the hypothesis that sample dissolution history impacts the measured dissolution rates in stacked experiments [8].

## 2. Materials and methods

The diopside used in this study is from Andhra Pradesh, India, and was obtained from Ward's Natural Science. Large crystals were crushed and 150–240 μm size fraction was used in all the experiments. The grains were washed ultrasonically in isopropanol to remove fine particles, rinsed repeatedly with deionized water and dried. The chemical composition of the mineral was determined using X-ray fluorescence and is given in Table 1. The stoichiometry of the diopside based on chemical composition is Ca_0.86_Mg_0.90_Fe_0.08_Al_0.03_Si_2.02_O_6_, when normalized to six oxygens. The BET specific surface area of the grains was 565 cm^2 ^g^-1^.

**Table 1:** Chemical composition of diopside.

Oxide	Wt%
SiO_2_	54.25
CaO	21.58
MgO	16.03
Fe_2_O_3_	3.01
Al_2_O_3_	0.61

All dissolution experiments were carried out in a titanium mixed flow-through reactor from Parr Instruments (see [20] for detailed description). A series of stacked experiments were performed by simply changing the input solution composition and/or the flow rate on the same mineral specimens to study mineral dissolution and precipitation kinetics as a function of solution composition without disturbing the mineral phase. The net dissolution rates normalized to their specific surface area (A) are calculated using the following expression

Ratenet=Δ[i]FRAυi     (1)
 MathType@MTEF@5@5@+=feaafiart1ev1aaatCvAUfKttLearuWrP9MDH5MBPbIqV92AaeXatLxBI9gBaebbnrfifHhDYfgasaacH8akY=wiFfYdH8Gipec8Eeeu0xXdbba9frFj0=OqFfea0dXdd9vqai=hGuQ8kuc9pgc9s8qqaq=dirpe0xb9q8qiLsFr0=vr0=vr0dc8meaabaqaciaacaGaaeqabaqabeGadaaakeaaieaacqWFsbGucqWFHbqycqWF0baDcqWFLbqzdaWgaaWcbaGae8NBa4Mae8xzauMae8hDaqhabeaakiabg2da9maalaaabaGaeyiLdqKaei4waSLae8xAaKMaeiyxa0Lae8NrayKae8NuaifabaGae8xqaeecciGae4xXdu3aaSbaaSqaaiab=LgaPbqabaaaaOGaaCzcaiaaxMaadaqadaqaaiabigdaXaGaayjkaiaawMcaaaaa@46E0@

where [*i*] is the difference between the effluent and influent concentration of a solute, FR is the flow rate, and υ_i _is the stoichiometric coefficient of the element *i *in the mineral formula. The experiments were conducted at an *in situ *pH of 7.5 and temperatures ranging from 125 to 175°C. The inlet solution was continuously purged with nitrogen to remove CO_2 _from the solution to avoid precipitation of carbonate minerals. About 2.5 grams of ground diopside were used in stacked experiments in 0.1 M NaCl solutions buffered using 20 mM sodium borate and HCl. Most of the stacked experiments approached equilibrium from high degrees of undersaturation by changing the flow rate from about 4 to 0.01 ml hr^-1^.

Experiments were also conducted to test the hypothesis that sample history can impact measured dissolution rates in stacked experiments. In one set of experiments equilibrium was approached from high degrees of undersaturation by decreasing the flow rate. In a second set of experiments, far from equilibrium conditions were approached from near equilibrium by decreasing the Ca concentration of the input solutions from 500 μM and then increasing the flow rate to obtain higher degrees of undersaturation. Solutions were analyzed for Ca, Mg, and Si by ICP-AES. Solution pH was measured at room temperature. The solution matrix of the standards was the same as the input solutions.

Aqueous speciation, ion activity, pH, and the Gibbs free energy of the reaction at elevated temperature were calculated using Geochemist's Workbench [21] by conducting a speciation calculation at 25°C based on room temperature measurements followed by a speciation calculation at the experiment temperature. Dissolution of diopside can be described by

CaMgSi_2_O_6 _+ 4H^+ ^+ 2H_2_O ⇔ Ca^2+ ^+ Mg^2+ ^+ 2H_4_SiO_4_.     (2)

The Gibbs free energy for the above dissolution reaction is calculated from

ΔGr=RTln⁡(aCa2+aMg2+aH4SiO42KeqaH+4)     (3)
 MathType@MTEF@5@5@+=feaafiart1ev1aaatCvAUfKttLearuWrP9MDH5MBPbIqV92AaeXatLxBI9gBaebbnrfifHhDYfgasaacH8akY=wiFfYdH8Gipec8Eeeu0xXdbba9frFj0=OqFfea0dXdd9vqai=hGuQ8kuc9pgc9s8qqaq=dirpe0xb9q8qiLsFr0=vr0=vr0dc8meaabaqaciaacaGaaeqabaqabeGadaaakeaacqGHuoarcqWGhbWrdaWgaaWcbaGaemOCaihabeaakiabg2da9iabdkfasjabdsfaujGbcYgaSjabc6gaUnaabmaabaWaaSaaaeaacqWGHbqydaWgaaWcbaGaem4qamKaemyyae2aaWbaaWqabeaacqaIYaGmcqGHRaWkaaaaleqaaOGaemyyae2aaSbaaSqaaiabd2eanjabdEgaNnaaCaaameqabaGaeGOmaiJaey4kaScaaaWcbeaakiabdggaHnaaDaaaleaacqWGibasdaWgaaadbaGaeGinaqdabeaaliabdofatjabdMgaPjabd+eapnaaBaaameaacqaI0aanaeqaaaWcbaGaeGOmaidaaaGcbaGaem4saS0aaSbaaSqaaiabdwgaLjabdghaXbqabaGccqWGHbqydaqhaaWcbaGaemisaG0aaWbaaWqabeaacqGHRaWkaaaaleaacqaI0aanaaaaaaGccaGLOaGaayzkaaGaaCzcaiaaxMaadaqadaqaaiabiodaZaGaayjkaiaawMcaaaaa@5A9A@

where, *K*_*eq *_is the equilibrium constant and *a*_*i *_represents the activity of the aqueous species. The equilibrium constants at 125, 150, 160, and 175°C are 10^14.48^, 10^13.27^, 10^12.82^, 10^12.19^, respectively [22]. No attempt was made to experimentally determine the equilibrium constant of the diopside in the study.

## 3. Results and Discussion

### 3.1. Steady-state concentration and stoichiometry of dissolution

**Figure 1:**
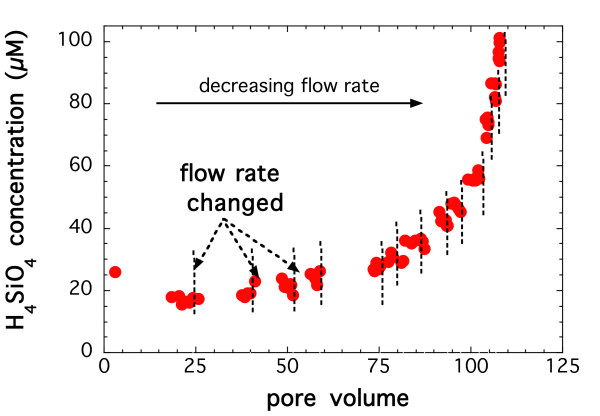
Silicic acid concentrations in the effluent as a function of residence volumes in a stacked experiment conducted at 150°C and *in situ *pH of 7.5. Steady-state conditions were assumed and the flow rates were changed (shown in vertical dashed lines) when the concentrations of the solutes in the effluent did not change with time.

An example of steady-state dissolution rates obtained in one of the stacked experiment conducted at 150°C is shown in Figure 1 by plotting the silicic acid concentrations in the effluent as a function of residence volumes, where the dashed lines indicate a change in flow rate. Steady-state conditions were assumed and the flow rates were changed when the concentrations of the solutes in the effluent did not change with time. At the highest flow rate, the concentration of silicic acid decreased with time and steady-state was achieved after about 20 reactor volumes. Steady-state was generally achieved in about 1–5 reactor volumes at lower flow rates. The steady-state concentrations reported for all the experiments were calculated as the average value of the final five samples where the concentrations in the effluent did not change with time.

**Figure 2:**
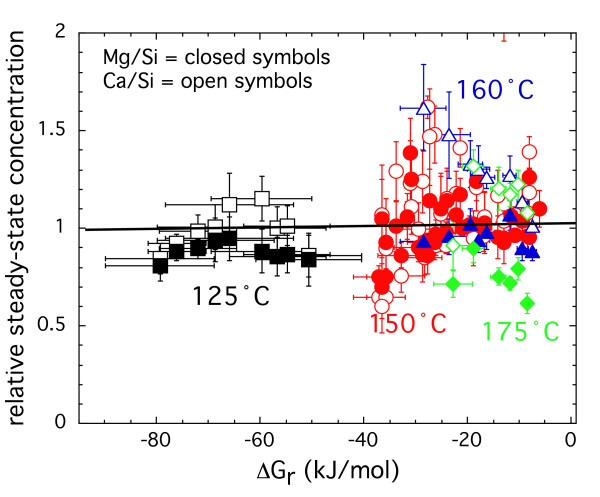
The ratio between Ca (open symbols) or Mg (closed symbols) concentration and Si concentration at steady-state divided by the stoichiometric number of moles of these elements in the solid is plotted against the Gibbs free energy of the reaction. The solid line indicates stoichiometric dissolution when the ratio equals 1. The color of the symbols and the indicated temperature are the same in the figure. All the experiments are conducted at an *in situ *pH of 7.5.

The steady-state Ca, Mg, and Si concentrations along with the flow rate are reported in Table 2 and illustrated in Figure 2 by plotting the ratio between Ca or Mg concentration and Si concentration at steady-state divided by the stoichiometric number of moles of these elements in the solid versus the Gibbs free energy of the reaction. In these plots, stoichiometric dissolution would be indicative when the ratio is close to 1. Diopside dissolution was congruent to within ± 0.4, with some higher and lower excursions. We do not attribute observed trends in the Ca to Si ratios at 160 and 175°C to preferential exchange of Ca over Mg from the surface or to precipitation of a secondary phase because the departure from congruent dissolution was on the same order as that observed for the much larger data set collected at 150°C. It is quite likely that that this trend would disappear with the collection of more rate data at 160 and 175°C. Net dissolution rates reported in this study from dissolved silica concentrations would increase by at most 0.2 log units at 160°C and 0.1 log units at 175°C if dissolved calcium concentrations were used to represent diopside dissolution. Similar small deviations from stoichiometric dissolution have been found for other minerals and may be in part be due to analytical uncertainty in both the solution and solid phase concentration of these elements (see [23] for a review).

**Table 2:** Results of diopside dissolution in flow-through experiments^1,2^.

Temp-ID	Si (μM)	Ca (μM)	Mg (μM)	pH(T)	Flow rate ml min^-1^	log Rate mol diospside cm^-2 ^s^-1^	ΔG_r _kJ mol^-1^	log⁡(aH+2aMg2+) MathType@MTEF@5@5@+=feaafiart1ev1aaatCvAUfKttLearuWrP9MDH5MBPbIqV92AaeXatLxBI9gBaebbnrfifHhDYfgasaacH8akY=wiFfYdH8Gipec8Eeeu0xXdbba9frFj0=OqFfea0dXdd9vqai=hGuQ8kuc9pgc9s8qqaq=dirpe0xb9q8qiLsFr0=vr0=vr0dc8meaabaqaciaacaGaaeqabaqabeGadaaakeaacyGGSbaBcqGGVbWBcqGGNbWzdaqadaqaamaalaaabaacbaGae8xyae2aa0baaSqaaiab=HeainaaCaaameqabaGaey4kaScaaaWcbaGaeGOmaidaaaGcbaGae8xyae2aaSbaaSqaaiab=1eanjab=DgaNnaaCaaameqabaGaeGOmaiJaey4kaScaaaWcbeaaaaaakiaawIcacaGLPaaaaaa@3D08@
175-1	26.8 ± 2.2	10.4 ± 1.2	8.5 ± 0.4	7.508	4.00	-12.20 ± 0.03	-22.78 ± 0.07	-9.35 ± 0.05
175-2	29.6 ± 1.0	16.6 ± 0.9	11.8 ± 0.9	7.513	2.00	-12.46 ± 0.02	-18.89 ± 0.05	-9.50 ± 0.02
175-3	43.6 ± 1.4	22.3 ± 1.9	14.6 ± 0.7	7.517	1.00	-12.59 ± 0.1	-14.01 ± 0.05	-9.60 ± 0.04
175-4	51.3 ± 1.6	25.6 ± 1.0	16.4 ± 0.6	7.520	0.50	-12.82 ± 0.1	-11.75 ± 0.03	-9.66 ± 0.02
175-5	54.8 ± 2.2	28.5 ± 1.3	19.3 ± 1.1	7.523	0.25	-13.10 ± 0.2	-10.15 ± 0.04	-9.73 ± 0.02
175-6	67.7 ± 1.5	31.1 ± 0.7	18.6 ± 1.6	7.523	0.10	-13.40 ± 0.1	-8.40 ± 0.04	-9.72 ± 0.01
160-1	19.0 ± 0.6	13.1 ± 1.8	7.9 ± 0.5	7.511	4.50	-12.30 ± 0.01	-28.48 ± 0.07	-9.36 ± 0.06
160-2	27.1 ± 2.2	17.0 ± 2.1	11.5 ± 0.5	7.515	2.00	-12.50 ± 0.04	-23.50 ± 0.08	-9.53 ± 0.05
160-3	35.8 ± 2.4	20.2 ± 1.2	16.2 ± 0.7	7.519	1.00	-12.68 ± 0.03	-19.50 ± 0.05	-9.69 ± 0.03
160-4	41.1 ± 2.3	22.8 ± 1.7	17.2 ± 0.6	7.520	0.75	-12.74 ± 0.02	-17.85 ± 0.05	-9.72 ± 0.03
160-5	45.4 ± 1.6	24.4 ± 0.7	19.8 ± 1.6	7.522	0.50	-12.88 ± 0.02	-16.30 ± 0.04	-9.78 ± 0.01
160-6	59.4 ± 3.4	32.2 ± 1.8	28.2 ± 1.2	7.53	0.25	-13.06 ± 0.02	-11.85 ± 0.05	-9.95 ± 0.02
160-7	75.6 ± 3.9	36.5 ± 2.3	30.2 ± 1.4	7.533	0.10	-13.35 ± 0.02	-9.32 ± 0.05	-9.99 ± 0.03
160-8	88.6 ± 4.5	38.2 ± 2.5	34.9 ± 0.6	7.536	0.05	-13.59 ± 0.02	-7.41 ± 0.04	-10.06 ± 0.03
150-A-1	16.8 ± 1.1	4.6 ± 0.4	5.6 ± 0.2	7.505	3.23	-12.50 ± 0.03	-37.00 ± 0.06	-9.23 ± 0.04
150-A-2	18.4 ± 0.8	5.1 ± 0.4	6.1 ± 0.2	7.506	2.37	-12.59 ± 0.02	-35.66 ± 0.05	-9.27 ± 0.04
150-A-3	20.9 ± 1.4	6.7 ± 0.6	8.0 ± 0.3	7.508	1.50	-12.74 ± 0.03	-32.79 ± 0.06	-9.39 ± 0.04
150-A-4	24.4 ± 1.7	10.3 ± 1.2	9.8 ± 0.4	7.511	1.00	-12.85 ± 0.03	-29.38 ± 0.07	-9.48 ± 0.05
150-A-5	27.3 ± 1.1	9.9 ± 1.0	11.3 ± 0.3	7.511	0.76	-12.92 ± 0.02	-28.23 ± 0.05	-9.54 ± 0.04
150-A-6	30.6 ± 1.3	11.9 ± 0.4	13.2 ± 0.7	7.513	0.51	-13.04 ± 0.02	-26.17 ± 0.04	-9.61 ± 0.01
150-A-7	35.4 ± 1.3	13.7 ± 0.3	15.4 ± 0.7	7.515	0.36	-13.13 ± 0.02	-24.04 ± 0.03	-9.69 ± 0.01
150-A-8	42.0 ± 1.0	17.9 ± 0.5	18.8 ± 0.4	7.519	0.22	-13.27 ± 0.01	-21.08 ± 0.02	-9.78 ± 0.01
150-A-9	47.0 ± 1.3	20.3 ± 0.8	21.3 ± 0.5	7.522	0.16	-13.36 ± 0.01	-19.66 ± 0.03	-9.80 ± 0.02
150-A-10	56.3 ± 1.3	23.0 ± 1.8	25.1 ± 0.7	7.525	0.10	-13.48 ± 0.01	-16.94 ± 0.04	-9.92 ± 0.03
150-A-11	73.3 ± 2.6	32.4 ± 1.2	30.9 ± 0.5	7.533	0.05	-13.67 ± 0.02	-12.90 ± 0.03	-10.02 ± 0.02
150-A-12	83.7 ± 2.6	38.1 ± 1.0	36.0 ± 0.8	7.538	0.03	-13.91 ± 0.01	-10.71 ± 0.02	-10.10 ± 0.01
150-A-13	97.2 ± 3.2	49.0 ± 3.6	41.4 ± 0.3	7.546	0.01	-14.25 ± 0.01	-8.03 ± 0.04	-10.18 ± 0.03
150-B-1	18.0 ± 1.4	4.6 ± 1.0	5.6 ± 1.2	7.505	4.00	-12.38 ± 0.03	-36.51 ± 0.14	-9.23 ± 0.09
150-B-2	19.8 ± 1.3	9.3 ± 1.0	11.0 ± 0.6	7.511	2.00	-12.64 ± 0.03	-30.83 ± 0.07	-9.53 ± 0.05
150-B-3	27.1 ± 1.7	17.1 ± 1.3	11.2 ± 0.8	7.515	1.00	-12.80 ± 0.03	-26.26 ± 0.06	-9.55 ± 0.03
150-B-4	36.7 ± 1.9	18.5 ± 1.5	17.5 ± 1.3	7.519	0.50	-12.97 ± 0.02	-22.18 ± 0.06	-9.75 ± 0.04
150-B-5	49.3 ± 1.4	19.9 ± 2.9	22.3 ± 1.3	7.522	0.10	-13.54 ± 0.01	-18.89 ± 0.07	-9.86 ± 0.06
150-B-6	86.7 ± 1.6	51.4 ± 2.8	48.5 ± 1.5	7.551	0.01	-14.30 ± 0.01	-7.96 ± 0.03	-10.26 ± 0.02
150-B-7	46.2 ± 3.2	23.7 ± 1.7	25.4 ± 1.0	7.526	0.10	-13.57 ± 0.03	-18.15 ± 0.06	-9.92 ± 0.03
150-B-8	36.1 ± 1.8	21.7 ± 1.1	18.8 ± 1.0	7.521	0.50	-12.98 ± 0.02	-21.41 ± 0.04	-9.78 ± 0.02
150-B-9	24.6 ± 0.7	17.0 ± 0.4	9.5 ± 1.0	7.514	1.00	-12.84 ± 0.01	-27.62 ± 0.05	-9.47 ± 0.01
150-B-10	16.4 ± 1.1	9.0 ± 0.9	7.3 ± 0.8	7.509	2.00	-12.72 ± 0.03	-33.77 ± 0.08	-9.35 ± 0.04
150-B-11	14.0 ± 2.1	6.4 ± 1.2	6.5 ± 0.8	7.507	4.00	-12.48 ± 0.06	-36.49 ± 0.13	-9.30 ± 0.08
150-C-1	32.2 ± 1.8	473.4 ± 10.3	15.8 ± 1.0	7.721	0.10	-13.73 ± 0.02	-5.97 ± 0.04	-10.11 ± 0.01
150-C-2	34.6 ± 1.0	283.6 ± 6.9	16.0 ± 1.0	7.646	0.10	-13.69 ± 0.01	-9.44 ± 0.04	-9.96 ± 0.01
150-C-3	42.8 ± 2.5	140.9 ± 6.3	20.3 ± 1.5	7.583	0.10	-13.60 ± 0.03	-11.48 ± 0.05	-9.94 ± 0.02
150-C-4	60.2 ± 3.7	54.7 ± 3.0	24.9 ± 2.5	7.541	0.10	-13.45 ± 0.03	-12.97 ± 0.06	-9.95 ± 0.02
150-C-5	65.4 ± 4.9	32.5 ± 2.8	27.8 ± 2.4	7.531	0.10	13.42 ± 0.03	-14.12 ± 0.07	-9.97 ± 0.04
150-C-6	32.3 ± 3.1	15.8 ± 1.5	16.4 ± 1.5	7.517	0.50	-13.03 ± 0.04	-23.91 ± 0.08	-9.72 ± 0.04
150-C-7	24.1 ± 2.6	15.1 ± 1.9	12.2 ± 1.1	7.515	1.00	-12.85 ± 0.05	-27.22 ± 0.10	-9.59 ± 0.06
150-C-8	17.8 ± 1.4	9.3 ± 1.5	11.0 ± 1.1	7.511	2.00	-12.68 ± 0.03	-31.07 ± 0.09	-9.59 ± 0.07
150-C-9	15.9 ± 1.6	6.1 ± 1.6	6.6 ± 0.7	7.507	4.00	-12.43 ± 0.04	-35.08 ± 0.14	-9.30 ± 0.11
150-C-10	19.8 ± 1.1	8.8 ± 0.6	9.3 ± 0.9	7.510	2.00	-12.64 ± 0.03	-31.65 ± 0.06	-9.46 ± 0.03
150-C-11	24.6 ± 2.9	13.0 ± 0.7	9.5 ± 1.0	7.512	1.00	-12.84 ± 0.05	-28.62 ± 0.09	-9.47 ± 0.02
150-C-12	30.6 ± 3.8	14.0 ± 1.8	15.0 ± 1.6	7.515	0.50	-13.05 ± 0.05	-25.10 ± 0.10	-9.67 ± 0.06
150-C-13	55.8 ± 5.2	25.1 ± 2.1	25.5 ± 3.0	7.526	0.10	-13.49 ± 0.04	-16.62 ± 0.08	-9.93 ± 0.04
150-C-14	96.7 ± 6.7	44.4 ± 2.2	41.3 ± 2.8	7.544	0.01	-14.25 ± 0.03	-8.49 ± 0.06	-10.17 ± 0.02
125-1	1.0 ± 0.1	0.4 ± 0.1	0.4 ± 0.1	7.500	4.50	-13.6 ± 0.02	-79.36 ± 0.06	-8.08 ± 0.03
125-2	1.3 ± 0.0	0.5 ± 0.1	0.5 ± 0.1	7.500	2.00	-13.8 ± 0.01	-76.09 ± 0.03	-8.21 ± 0.02
125-3	1.7 ± 0.1	0.7 ± 0.1	0.7 ± 0.1	7.501	1.00	-14.0 ± 0.02	-72.07 ± 0.05	-8.34 ± 0.03
125-4	2.1 ± 0.1	0.9 ± 0.1	0.9 ± 0.1	7.501	0.50	-14.2 ± 0.01	-68.60 ± 0.07	-8.47 ± 0.05
125-5	2.5 ± 0.1	1.2 ± 0.1	1.1 ± 0.1	7.501	0.25	-14.4 ± 0.05	-65.99 ± 0.08	-8.54 ± 0.04
125-6	4.1 ± 0.1	2.0 ± 0.1	1.6 ± 0.1	7.502	0.10	-14.6 ± 0.04	-59.72 ± 0.07	-8.72 ± 0.02
125-7	5.4 ± 0.1	2.3 ± 0.1	2.0 ± 0.1	7.502	0.05	-14.8 ± 0.03	-56.63 ± 0.07	-8.83 ± 0.04
125-8	6.2 ± 0.1	2.7 ± 0.1	2.4 ± 0.1	7.503	0.025	-15.0 ± 0.03	-54.78 ± 0.06	-8.90 ± 0.03
125-9	8.8 ± 0.1	3.2 ± 0.1	3.3 ± 0.1	7.504	0.01	-15.3 ± 0.05	-50.61 ± 0.09	-9.04 ± 0.03

^1 ^The sequence of a given stacked experiment is indicated in the Temperature-ID.

^2 ^pH(T) was calculated by charge balance from measured solution composition.

**Figure 3:**
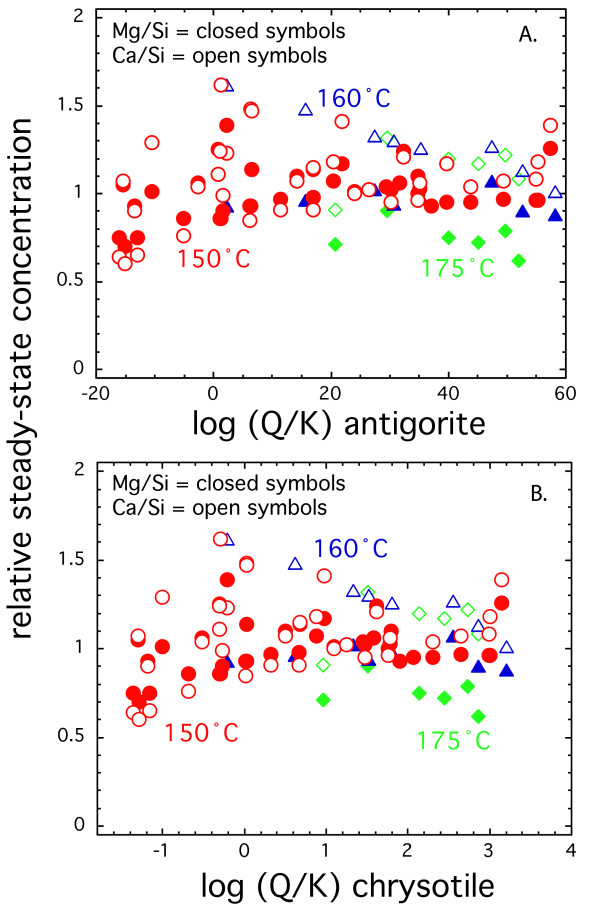
Stoichiometric ratio of Ca (open symbols) or Mg (closed symbols) with respect to Si at steady-state versus saturation with respect to (A) antigorite and (B) chrysotile. Saturation state was calculated based on the steady-state solute concentrations using Supcrit92 database [22]. The color of the symbols and the indicated temperature are the same in the figure.

The effluent solute concentrations were supersaturated with respect to antigorite (Mg_3_Si_2_O_5_) and chrysotile (Mg_3_Si_2_O_5_(OH)_4_) under most conditions above 150°C (Figure 3). Mg-silicates exhibit retrograde solubility (i.e., a solubility decrease with increasing temperature). Therefore, precipitation of these minerals is favored at higher temperature for similar effluent concentrations. Precipitation of Mg-rich silicate minerals during the experiments should significantly lower the stoichiometric ratio of Mg/Si, because these phases have three times Mg relative to Si in diopside. Such large lowering in the Mg/Si ratio was not observed at any of the temperatures.

### 3.2. Hysteresis in dissolution rates as a function of saturation state

A chief advantage of using mixed flow-through reactors to study mineral dissolution and precipitation kinetics is that it allows the rate at which minerals dissolve and precipitate to be evaluated as a function of solution composition without disturbing the mineral phase. As a result, experiments are typically performed in series of stacked experiments by simply changing the input solution composition and/or the flow rate on the same mineral specimens. Beig and Luttge [8] raised the concern that stacked experiments started from high or low degree of undersaturation can have a major impact on the observed rate dependency on solution saturation state, and hence the mechanisms invoked to explain the dissolution behavior. Beig and Luttge [8]compared dissolution rates for albite (NaAlSi_3_O_8_) initially treated at 185°C and pH 9 with an output solution composition that was far from equilibrium (ΔG_r _< 35 kJ/mol) with dissolution rates of untreated albite surfaces. When the treated and untreated specimens were subsequently reacted at the same conditions, they found that the treated albite dissolution rates were 0.6 to 2 orders of magnitude faster than the untreated samples depending on the solution composition; the difference in rates were higher closer to equilibrium. The authors showed that the faster dissolution rates of the treated samples occurred on pre-existing etch pits from the initial treatment and at step edges, while the slower dissolution rates of untreated samples occurred mostly at step edges.

**Figure 4:**
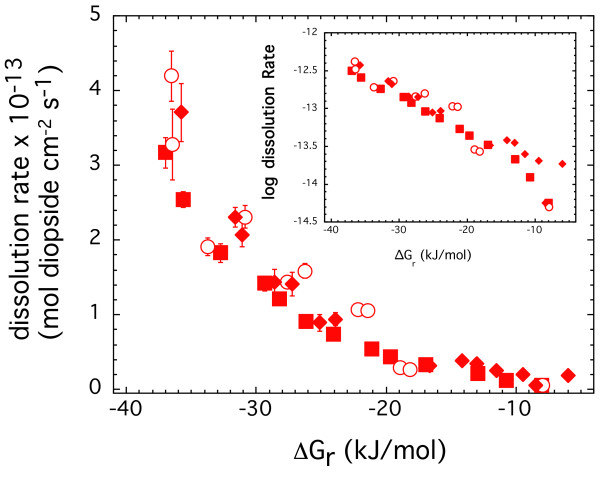
Diopside dissolution rates measured at 150°C and *in situ *pH of 7.5 in stacked experiments starting at far from (squares and circles) and close to (diamonds) equilibrium conditions are plotted against the Gibbs free energy of the reaction. The rates in the insert are plotted as logarithm of rates to highlight the differences in the measured rates observed at close to equilibrium conditions.

Diopside dissolution rates measured at 150°C in stacked experiments starting at far and close to equilibrium conditions are shown in Figure 4. The differences in rates at small reaction affinity are highlighted in the insert plotted as log dissolution rate versus the Gibbs free energy of reaction. The rates were at most 3 times higher at the maximum ΔG_r _compared to diopside dissolution in stacked experiments started at far from equilibrium conditions. The difference in rates or hysteresis quickly diminishes in more undersaturated solutions. Dissolution rates obtained in all the experiments were similar to each other when ΔG_r _< -12 kJ/mol, regardless of the initial starting conditions of the stacked experiments. Based on these results, we conclude that experimental protocol does not significantly impact dissolution rates measured over a range saturation states for ground diopside specimens.

### 3.3. Dissolution rate as a function of ΔG_r_

A generalized rate law for overall mineral dissolution can be written as

**Figure 5:**
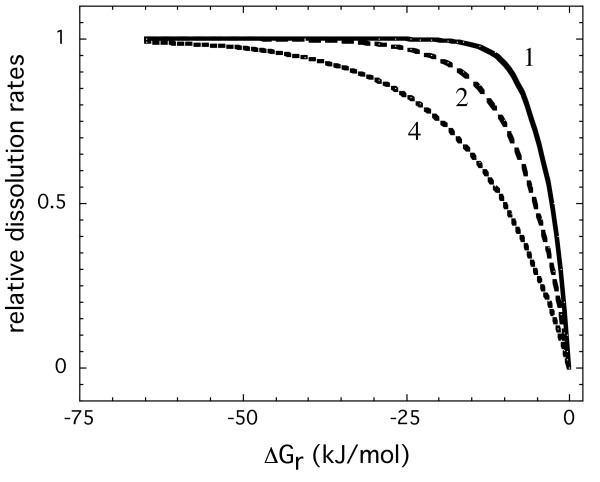
Predicted rates, normalized to the maximum rate, as a function of the Gibbs free energy of the reaction for different Temkin's co-efficient (indicated by the numbers shown in the figure close to the lines).

R_diss _= *k*_+ _*f*(ΔG_r_),     (4)

where ΔG_r _= RT lnΩ = RT ln(Q/K_eq_), Ω is the saturation state, Q and K_eq _are the ion activity quotient and equilibrium constant of the dissolution reaction, respectively, and *k*_+ _is the apparent rate constant for the forward reaction at a given temperature which may include the effect of pH, presence of other solutes which might inhibit or enhance dissolution, and reactive surface area. The functional dependence of the rate on the Gibbs free energy of reaction (ΔG_r_) has been derived from transition state theory and, in its simplest form, is given by [24].

f(ΔGr)=1−exp⁡(ΔGrσRT)     (5)
 MathType@MTEF@5@5@+=feaafiart1ev1aaatCvAUfKttLearuWrP9MDH5MBPbIqV92AaeXatLxBI9gBaebbnrfifHhDYfgasaacH8akY=wiFfYdH8Gipec8Eeeu0xXdbba9frFj0=OqFfea0dXdd9vqai=hGuQ8kuc9pgc9s8qqaq=dirpe0xb9q8qiLsFr0=vr0=vr0dc8meaabaqaciaacaGaaeqabaqabeGadaaakeaacqWGMbGzcqGGOaakcqGHuoarieaacqWFhbWrdaWgaaWcbaGae8NCaihabeaakiabcMcaPiabg2da9iabigdaXiabgkHiTiGbcwgaLjabcIha4jabcchaWnaabmaabaWaaSaaaeaacqGHuoarcqWFhbWrdaWgaaWcbaGae8NCaihabeaaaOqaaGGaciab+n8aZjab=jfasjab=rfaubaaaiaawIcacaGLPaaacaWLjaGaaCzcamaabmaabaGaeGynaudacaGLOaGaayzkaaaaaa@4882@

where, *σ *is the stoichiometric number of moles of precursor complex formed from one mole of the mineral also known as the Temkin's co-efficient, R is the gas constant, and T the absolute temperature. Temkin's co-efficient values of 1 to 3 have been used to describe macroscopic dissolution rate data [15]. With the above formulation, a dissolution plateau should be observed at conditions far from equilibrium when dissolution rates are largely independent of saturation, followed by a very strong dependence on saturation very close to equilibrium (Figure 5). At a fixed temperature, the decrease in rate as equilibrium is approached is largely sensitive to the Temkin's co-efficient where the rate becomes more dependent on ΔG_r _at conditions farther from equilibrium at higher values of the Temkin's co-efficient. Although this simple dependence of dissolution rate on ΔG_r _can be applied to a wide range of mineral systems, it has been observed only for quartz and silica polymorphs in dilute simple electrolyte solutions [7,9,25-27]. For other silicates and aluminum bearing minerals, dissolution behavior is more complex [2-20]. This is also the case for diopside.

**Figure 6:**
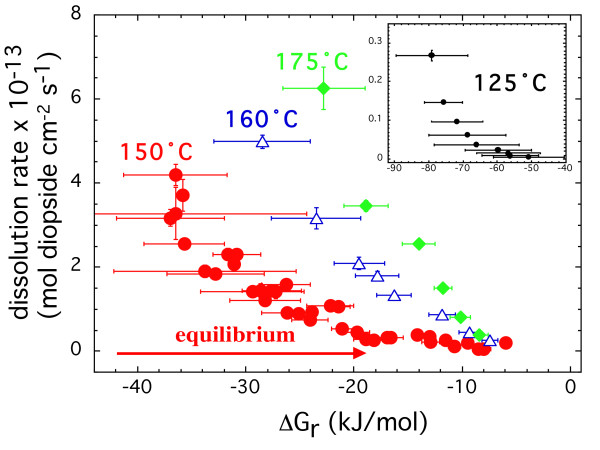
Steady-state dissolution rate of diopside measured at different temperatures are plotted as a function of Gibbs free energy of the dissolution reaction. The insert is for data collected at 125°C. All the experiments were conducted at an *in situ *pH of 7.5. The temperature of the experiment is indicated with the same color as the data.

Dissolution rate of diopside as a function of ΔG_r _at all the temperatures is shown in Figure 6. Diopside dissolution rates continuously decreased with increasing ΔG_r _and cannot be attributed solely to the degree of undersaturation (equation 5). None of the trends display a dissolution plateau, when rates are independent of ΔG_r _The trend in rates shows a convex dependence on ΔG_r_, and not the concave dependence that would result from higher Temkin's coefficient values. Rates generally decreased by two orders of magnitude when ΔG_r _is increased from -40 to -5 kJ mol^-1 ^at 150, 160 and 175°C. Even at 125°C, where rates were collected at much lower ΔG_r _rates decreased by two orders of magnitude when ΔG_r _was increased from -80 to -50 kJ mol^-1^. It is possible that diopside dissolution may display a dissolution plateau at lower ΔG_r _values than those studied here, because dissolution plateaus for various minerals have been observed at different degrees of undersaturation [2-6,8,10,11,14,15,19]. For example, dissolution plateaus are observed at relatively high ΔG_r _values for gibbsite (-5 kJ/mol), intermediate values for albite and labradorite (-50 kJ/mol), and low values for K-feldspar and smectite (>-80 kJ/mol).

An extension of transition state theory where a rate limiting ion exchange reaction controls dissolution [15-18] and an extension of crystal growth theory to dissolution dominated by 2D nucleation of etch pits or by detachment of ions at dislocation sites [7] have been used to explain similar continuous decreases in dissolution rates with approach to equilibrium. We generally refer to these models as the ion exchange and pit nucleation models. Below we use diopside dissolution rates that span over three orders of magnitude, a wide range of ΔG_r _and temperature to evaluate these two models which propose distinct dissolution mechanisms. We also derive corresponding rate expressions, because an important strength of both of these models is that rates are linked to solution saturation allowing complex description of geochemical processes when kinetic and thermodynamic data bases are coupled with flow and transport.

## 4. Ion Exchange Model

Oelkers [18] expanded equation 4 to explicitly account for the dependence of multi-oxide silicate mineral dissolution rates on solution composition by the formation of rate-limiting Si-rich surface complexes formed by metal-proton exchange reactions. The hydrolysis of the Si-O-Si bonds ultimately results in the dissolution of the mineral. These authors also note that for some framework silicate minerals the mineral is dissolved only through metal-proton exchange reactions. This model has been used to describe the dependence of alumino-silicate minerals on dissolved aluminum concentrations and the dependence of magnesio-silicate minerals and glass on dissolved magnesium concentrations. For alumino-silicate minerals, alkali and alkaline earth metals are exchanged fast and the Si-rich surface precursor complexes are formed from Al-H exchange reactions [11,15,16,20]. For mafic silicates, Oelkers (2001) predicts that the Ca-H exchange reaction will precede Mg-H exchange reaction and that rate-limiting Si-rich surface precursor complexes are formed by Mg-H exchange [13,15]. The concentration of the surface complexes would be therefore dependent on the dissolved Mg and pH according to the following reaction:

>*n*MgSiO + 2*n*H^+ ^= >SiOH_2*n *_+ *n*Mg^2+^,     (6)

where, *n *is the stoichiometric exchange coefficient for H^+ ^and Mg^2+^, >*n*MgSiO and >SiOH_2*n *_are the Mg-filled and the Si-rich mineral surface sites. Using transition state theory and assuming that the forward rate of the dissolution of minerals is proportional to the concentration of the Si-rich surface complex, and that there is a fixed number of mineral surface sites, the net dissolution rate of diopside is then given by

Rnet=k+⌊(aH+2aMg2+)nK1+K(aH+2aMg2+)n⌋[1−exp⁡(ΔGrσRT)]     (7)
 MathType@MTEF@5@5@+=feaafiart1ev1aaatCvAUfKttLearuWrP9MDH5MBPbIqV92AaeXatLxBI9gBaebbnrfifHhDYfgasaacH8akY=wiFfYdH8Gipec8Eeeu0xXdbba9frFj0=OqFfea0dXdd9vqai=hGuQ8kuc9pgc9s8qqaq=dirpe0xb9q8qiLsFr0=vr0=vr0dc8meaabaqaciaacaGaaeqabaqabeGadaaakeaaieaacqWFsbGudaWgaaWcbaGae8NBa4Mae8xzauMae8hDaqhabeaakiabg2da9iabdUgaRnaaBaaaleaacqGHRaWkaeqaaOWaayWaaeaadaWcaaqaamaabmaabaWaaSaaaeaacqWFHbqydaqhaaWcbaGae8hsaG0aaWbaaWqabeaacqGHRaWkaaaaleaacqaIYaGmaaaakeaacqWFHbqydaWgaaWcbaGae8xta0Kae83zaC2aaWbaaWqabeaacqaIYaGmcqGHRaWkaaaaleqaaaaaaOGaayjkaiaawMcaamaaCaaaleqabaGae8NBa4gaaOGaem4saSeabaGaeGymaeJaey4kaSIaem4saS0aaeWaaeaadaWcaaqaaiab=fgaHnaaDaaaleaacqWFibasdaahaaadbeqaaiabgUcaRaaaaSqaaiabikdaYaaaaOqaaiab=fgaHnaaBaaaleaacqWFnbqtcqWFNbWzdaahaaadbeqaaiabikdaYiabgUcaRaaaaSqabaaaaaGccaGLOaGaayzkaaWaaWbaaSqabeaacqWFUbGBaaaaaaGccaGLWJVaay5+4dWaamWaaeaacqaIXaqmcqGHsislcyGGLbqzcqGG4baEcqGGWbaCdaqadaqaamaalaaabaGaeyiLdqKae83raC0aaSbaaSqaaiab=jhaYbqabaaakeaaiiGacqGFdpWCcqWFsbGucqWFubavaaaacaGLOaGaayzkaaaacaGLBbGaayzxaaGaaCzcaiaaxMaadaqadaqaaiabiEda3aGaayjkaiaawMcaaaaa@701D@

where *k*_+ _is the apparent forward dissolution rate constant and *K *is the equilibrium constant for the formation of the Si-rich surface complex (Equation 6). When relatively low concentrations of the surface precursor complex are present such that K(aH+2aMg2+)n
 MathType@MTEF@5@5@+=feaafiart1ev1aaatCvAUfKttLearuWrP9MDH5MBPbIqV92AaeXatLxBI9gBaebbnrfifHhDYfgasaacH8akY=wiFfYdH8Gipec8Eeeu0xXdbba9frFj0=OqFfea0dXdd9vqai=hGuQ8kuc9pgc9s8qqaq=dirpe0xb9q8qiLsFr0=vr0=vr0dc8meaabaqaciaacaGaaeqabaqabeGadaaakeaaieaacqWFlbWsdaqadaqaamaalaaabaGae8xyae2aa0baaSqaaiab=HeainaaCaaameqabaGaey4kaScaaaWcbaGaeGOmaidaaaGcbaGae8xyae2aaSbaaSqaaiab=1eanjab=DgaNnaaCaaameqabaGaeGOmaiJaey4kaScaaaWcbeaaaaaakiaawIcacaGLPaaadaahaaWcbeqaaiab=5gaUbaaaaa@3B93@ is substantially less than 1, then dissolution rates are dependent on the activity of H^+ ^and Mg^2+ ^and equation 7 reduces to

Rnet=k(aH+2aMg2+)n     (8)
 MathType@MTEF@5@5@+=feaafiart1ev1aaatCvAUfKttLearuWrP9MDH5MBPbIqV92AaeXatLxBI9gBaebbnrfifHhDYfgasaacH8akY=wiFfYdH8Gipec8Eeeu0xXdbba9frFj0=OqFfea0dXdd9vqai=hGuQ8kuc9pgc9s8qqaq=dirpe0xb9q8qiLsFr0=vr0=vr0dc8meaabaqaciaacaGaaeqabaqabeGadaaakeaaieaacqWFsbGudaWgaaWcbaGae8NBa4Mae8xzauMae8hDaqhabeaakiabg2da9iabdUgaRnaabmaabaWaaSaaaeaacqWFHbqydaqhaaWcbaGae8hsaG0aaWbaaWqabeaacqGHRaWkaaaaleaacqaIYaGmaaaakeaacqWFHbqydaWgaaWcbaGae8xta0Kae83zaC2aaWbaaWqabeaacqaIYaGmcqGHRaWkaaaaleqaaaaaaOGaayjkaiaawMcaamaaCaaaleqabaGae8NBa4gaaOGaaCzcaiaaxMaadaqadaqaaiabiIda4aGaayjkaiaawMcaaaaa@462E@

where *k *= *k*_+_*K*. Under these conditions, the relation between log R_net _and log⁡(aH+2aMg2+)
 MathType@MTEF@5@5@+=feaafiart1ev1aaatCvAUfKttLearuWrP9MDH5MBPbIqV92AaeXatLxBI9gBaebbnrfifHhDYfgasaacH8akY=wiFfYdH8Gipec8Eeeu0xXdbba9frFj0=OqFfea0dXdd9vqai=hGuQ8kuc9pgc9s8qqaq=dirpe0xb9q8qiLsFr0=vr0=vr0dc8meaabaqaciaacaGaaeqabaqabeGadaaakeaacyGGSbaBcqGGVbWBcqGGNbWzdaqadaqaamaalaaabaacbaGae8xyae2aa0baaSqaaiab=HeainaaCaaameqabaGaey4kaScaaaWcbaGaeGOmaidaaaGcbaGae8xyae2aaSbaaSqaaiab=1eanjab=DgaNnaaCaaameqabaGaeGOmaiJaey4kaScaaaWcbeaaaaaakiaawIcacaGLPaaaaaa@3D08@ is linear and *n *is represented by the slope and log *k *is given as the y-intercept.

**Figure 7:**
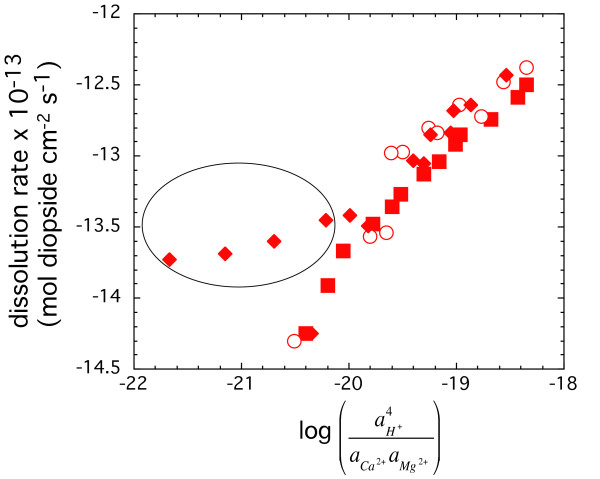
Logarithm of the steady-state dissolution rates as a function of log⁡(aH+4aCa2+aMg2+)
 MathType@MTEF@5@5@+=feaafiart1ev1aaatCvAUfKttLearuWrP9MDH5MBPbIqV92AaeXatLxBI9gBaebbnrfifHhDYfgasaacH8akY=wiFfYdH8Gipec8Eeeu0xXdbba9frFj0=OqFfea0dXdd9vqai=hGuQ8kuc9pgc9s8qqaq=dirpe0xb9q8qiLsFr0=vr0=vr0dc8meaabaqaciaacaGaaeqabaqabeGadaaakeaacyGGSbaBcqGGVbWBcqGGNbWzdaqadaqaamaalaaabaacbaGae8xyae2aa0baaSqaaiab=HeainaaCaaameqabaGaey4kaScaaaWcbaGaeGinaqdaaaGcbaGae8xyae2aaSbaaSqaaiab=neadjab=fgaHnaaCaaameqabaGaeGOmaiJaey4kaScaaaWcbeaakiab=fgaHnaaBaaaleaacqWFnbqtcqWFNbWzdaahaaadbeqaaiabikdaYiabgUcaRaaaaSqabaaaaaGccaGLOaGaayzkaaaaaa@42E8@. The experiments are conducted at 150°C and *in situ *pH of 7.5. The symbols represent different stacked experiments: squares and circles represent experiments initiated at far from equilibrium conditions, whereas, the symbols represented by diamonds were started at close to equilibrium conditions. The data points shown inside the oval were initiated with Ca in the input solution and were started at close to equilibrium conditions.

The formation of Si-rich surface complexes could also be described as a function of both Ca-H and Mg-H exchange on the diopside surface. However, we model diopside dissolution as being limited by the concentration of Si-rich precursor complexes formed by Mg-H based on the few experiments conducted with excess Ca in the input solution (Figure 7). Dissolution rates in experiments with excess Ca were nearly independent of log⁡(aH+2aca2+aMg2+)
 MathType@MTEF@5@5@+=feaafiart1ev1aaatCvAUfKttLearuWrP9MDH5MBPbIqV92AaeXatLxBI9gBaebbnrfifHhDYfgasaacH8akY=wiFfYdH8Gipec8Eeeu0xXdbba9frFj0=OqFfea0dXdd9vqai=hGuQ8kuc9pgc9s8qqaq=dirpe0xb9q8qiLsFr0=vr0=vr0dc8meaabaqaciaacaGaaeqabaqabeGadaaakeaacyGGSbaBcqGGVbWBcqGGNbWzdaqadaqaamaalaaabaacbaGae8xyae2aa0baaSqaaiabdIeainaaCaaameqabaGaey4kaScaaaWcbaGaeGOmaidaaaGcbaGae8xyae2aaSbaaSqaaiabdogaJjabdggaHnaaCaaameqabaGaeGOmaiJaey4kaScaaaWcbeaakiab=fgaHnaaBaaaleaacqWGnbqtcqWGNbWzdaahaaadbeqaaiabikdaYiabgUcaRaaaaSqabaaaaaGccaGLOaGaayzkaaaaaa@4338@ compared to the strong dependence of dissolution rate on log⁡(aH+2aca2+aMg2+)
 MathType@MTEF@5@5@+=feaafiart1ev1aaatCvAUfKttLearuWrP9MDH5MBPbIqV92AaeXatLxBI9gBaebbnrfifHhDYfgasaacH8akY=wiFfYdH8Gipec8Eeeu0xXdbba9frFj0=OqFfea0dXdd9vqai=hGuQ8kuc9pgc9s8qqaq=dirpe0xb9q8qiLsFr0=vr0=vr0dc8meaabaqaciaacaGaaeqabaqabeGadaaakeaacyGGSbaBcqGGVbWBcqGGNbWzdaqadaqaamaalaaabaacbaGae8xyae2aa0baaSqaaiabdIeainaaCaaameqabaGaey4kaScaaaWcbaGaeGOmaidaaaGcbaGae8xyae2aaSbaaSqaaiabdogaJjabdggaHnaaCaaameqabaGaeGOmaiJaey4kaScaaaWcbeaakiab=fgaHnaaBaaaleaacqWGnbqtcqWGNbWzdaahaaadbeqaaiabikdaYiabgUcaRaaaaSqabaaaaaGccaGLOaGaayzkaaaaaa@4338@ from experiments conducted in the absence of excess Ca. This comparison suggests that for diopside the precursor complex is formed by Mg-H exchange.

**Figure 8:**
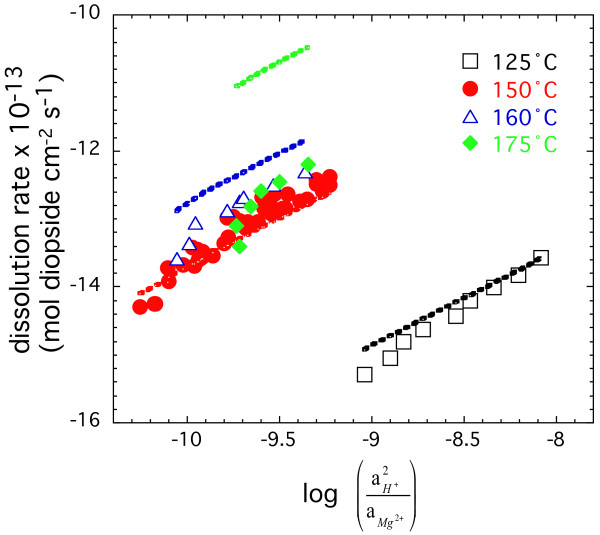
Logarithm of the steady-state dissolution rates as a function of log⁡(aH+2aMg2+)
 MathType@MTEF@5@5@+=feaafiart1ev1aaatCvAUfKttLearuWrP9MDH5MBPbIqV92AaeXatLxBI9gBaebbnrfifHhDYfgasaacH8akY=wiFfYdH8Gipec8Eeeu0xXdbba9frFj0=OqFfea0dXdd9vqai=hGuQ8kuc9pgc9s8qqaq=dirpe0xb9q8qiLsFr0=vr0=vr0dc8meaabaqaciaacaGaaeqabaqabeGadaaakeaacyGGSbaBcqGGVbWBcqGGNbWzdaqadaqaamaalaaabaacbaGae8xyae2aa0baaSqaaiab=HeainaaCaaameqabaGaey4kaScaaaWcbaGaeGOmaidaaaGcbaGae8xyae2aaSbaaSqaaiab=1eanjab=DgaNnaaCaaameqabaGaeGOmaiJaey4kaScaaaWcbeaaaaaakiaawIcacaGLPaaaaaa@3D08@. The symbols represent experimentally determined rates obtained at different temperatures. The data were fitted with an ion exchange model and the fitted rates are shown in the figure by colored lines. The same color is used for the experimentally determined and fitted rates (see text for details).

Figure 8 shows that the dependence of diopside dissolution on solution composition at 125, 150, 160, and 175°C can be described by the formation of Si-rich surface complexes by Mg-H exchange as is shown in plots of log R_net _versus log⁡(aH+2aMg2+)
 MathType@MTEF@5@5@+=feaafiart1ev1aaatCvAUfKttLearuWrP9MDH5MBPbIqV92AaeXatLxBI9gBaebbnrfifHhDYfgasaacH8akY=wiFfYdH8Gipec8Eeeu0xXdbba9frFj0=OqFfea0dXdd9vqai=hGuQ8kuc9pgc9s8qqaq=dirpe0xb9q8qiLsFr0=vr0=vr0dc8meaabaqaciaacaGaaeqabaqabeGadaaakeaacyGGSbaBcqGGVbWBcqGGNbWzdaqadaqaamaalaaabaacbaGae8xyae2aa0baaSqaaiab=HeainaaCaaameqabaGaey4kaScaaaWcbaGaeGOmaidaaaGcbaGae8xyae2aaSbaaSqaaiab=1eanjab=DgaNnaaCaaameqabaGaeGOmaiJaey4kaScaaaWcbeaaaaaakiaawIcacaGLPaaaaaa@3D08@. The trends are highly linear and indicate a minimal effect of solution saturation even at ΔG_r _close to equilibrium. We fit our data by multiple linear regression to an expanded form of equation 8 to describe diopside dissolution as a function of temperature as well as solution composition:

Rate (moldiopsidecm−2s−1)=k×10−Ea/2.303RT(aH+2aMg2+)n
 MathType@MTEF@5@5@+=feaafiart1ev1aaatCvAUfKttLearuWrP9MDH5MBPbIqV92AaeXatLxBI9gBaebbnrfifHhDYfgasaacH8akY=wiFfYdH8Gipec8Eeeu0xXdbba9frFj0=OqFfea0dXdd9vqai=hGuQ8kuc9pgc9s8qqaq=dirpe0xb9q8qiLsFr0=vr0=vr0dc8meaabaqaciaacaGaaeqabaqabeGadaaakeaaieaacqWFsbGucqWFHbqycqWF0baDcqWFLbqzcqqGGaaicqGGOaakcqWFTbqBcqWFVbWBcqWFSbaBcqWFGaaicqWFKbazcqWFPbqAcqWFVbWBcqWFWbaCcqWFZbWCcqWFPbqAcqWFKbazcqWFLbqzcqWFGaaicqWFJbWycqWFTbqBdaahaaWcbeqaaiabgkHiTiabikdaYaaakiab=bcaGiab=nhaZnaaCaaaleqabaGaeyOeI0IaeGymaedaaOGaeiykaKIaeyypa0Jaem4AaSMaey41aqRaeeymaeJaeeimaaZaaWbaaSqabeaacqGHsislcqWGfbqrdaWgaaadbaGaemyyaegabeaaliabc+caViabikdaYiabc6caUiabiodaZiabicdaWiabiodaZiabdkfasjabdsfaubaakmaabmaabaWaaSaaaeaacqWFHbqydaqhaaWcbaGaemisaG0aaWbaaWqabeaacqGHRaWkaaaaleaacqaIYaGmaaaakeaacqWFHbqydaWgaaWcbaGaemyta0Kaem4zaC2aaWbaaWqabeaacqaIYaGmcqGHRaWkaaaaleqaaaaaaOGaayjkaiaawMcaamaaCaaaleqabaGaemOBa4gaaaaa@6D9A@     (9)

We do not fit the data at 175°C, because diopside dissolution rates are of similar magnitude at 175 and 160°C indicating secondary mineral precipitation at 175°C. It seems unlikely that the fall off in rates represents a leveling off of the activation energy at higher temperatures, because *E*_*a *_typically increases with temperature for mineral systems [28]. Nor is it likely that the fall off in rates represents a change in mechanism due to a more alkaline pH at higher temperature. The solution OH^- ^concentrations are similar based on a minimal decrease in pK_w _of only 0.1 log units between 160 and 175°C [22]. The best fit to the data was obtained with *n *= 1.39, *E*_*a *_= 332 (kJ mol^-1^) and *k *= 10^41.2 ^(mol diopside cm^-2 ^s^-1^). A comparison between the experimental data and the fitted values, with an extrapolation to 175°C, are shown in Figure 8. The ion exchange model adequately describes diopside dissolution to within 0.5 log units from 125 to 160°C. Extrapolation of this model to 175°C suggests that the net measured rate is offset by precipitation of a secondary phase that is about 1.5 to 2.0 log units higher the net measured dissolution rate. It appears that the secondary precipitate is a Ca-Mg-silicate rather than a Mg-silicate, because the difference of rates calculated from dissolve Ca (which is nominally undersaturated with mineral phases) and dissolved Mg and Si concentrations do not account for difference between observation and model. Fits did not improve when ΔG_r_, *K *and an associated enthalpy term were included to describe the full form of the ion exchange model.

The apparent activation energy obtained in this study is much higher than those reported previously, which varied from about 40 to 150 kJ mol^-1 ^[29-32]. It is possible that the much higher activation energy reported may be due to differences in rate models and the temperature range studied. Previous studies did not explicitly account for the effect of solution saturation as was done here with the ion exchange model. The net result would be a lower activation energy derived from averaged rate constants. The previous studies were also conducted at temperatures below 100°C, where the activation energy may be lower.

## 5. Pit Nucleation Model

Dissolution mechanisms and rates have been explained recently using theories developed previously for crystal growth [33,34]. Extension of crystal growth theory to mineral dissolution calls for dissolution through retreat of steps, whose velocity (ν) is dependent on the solution saturation state (Ω) by the following expression

ν = ωβK_eq_(Ω-1)     (10)

where β is the step kinetic co-efficient, ω is the molar volume of a molecule in the crystal, and K_eq _is the equilibrium constant of the dissolution reaction. These steps originate from dislocations within the mineral crystal as pre-existing features or develop by nucleation of two-dimensional pits in an otherwise perfect surface once the energy barrier to their formation is overcome. Dissolution rates depend on the step source and density. In this paper we focus on dissolution controlled by homogeneous and defect-assisted nucleation, because they appear to be the dominant mechanisms for diopside over step retreat at dislocations [7]. The dissolution by nucleation of two-dimensional pits can be initiated in an otherwise perfect surface only if the free energy barrier to the formation of a pit is overcome. The resulting free energy is given by

ΔGcrit=−πa2ωhkTlnΩ     (11)
 MathType@MTEF@5@5@+=feaafiart1ev1aaatCvAUfKttLearuWrP9MDH5MBPbIqV92AaeXatLxBI9gBaebbnrfifHhDYfgasaacH8akY=wiFfYdH8Gipec8Eeeu0xXdbba9frFj0=OqFfea0dXdd9vqai=hGuQ8kuc9pgc9s8qqaq=dirpe0xb9q8qiLsFr0=vr0=vr0dc8meaabaqaciaacaGaaeqabaqabeGadaaakeaacqGHuoarieaacqWFhbWrdaWgaaWcbaGae83yamMae8NCaiNae8xAaKMae8hDaqhabeaakiabg2da9iabgkHiTmaalaaabaacciGae4hWdaNaemyyae2aaWbaaSqabeaaieGacqqFYaGmaaGccqGFjpWDcqWGObaAaeaaieqacqaFRbWAcqWGubavcqqFSbaBcqqFUbGBcqqHPoWvaaGaaCzcaiaaxMaadaqadaqaaiabigdaXiabigdaXaGaayjkaiaawMcaaaaa@49BF@

where α is the step edge free energy, *h *is the step height, **k **the Boltzmann constant. As equation 11 predicts, the free energy barrier is dependent on temperature, degree of undersaturation, and by factors that affect the step edge free energy. According to this model, dissolution rates would then decrease continuously as equilibrium is approached because the number of pits decreases with decreasing reaction affinity. Additionally, homogeneous nucleation of pits should transition to defect-assisted nucleation of pits at conditions closer to equilibrium. The dependence of dissolution rates originating from nucleation of pits on the degree of undersaturation is then given by

R = *h*(υ^2^J)^1/3 ^    (12)

where *h *is the step height and *J *is the nucleation rate. The steady-state nucleation rate is derived from nucleation theory and is given by

J=|1ln⁡Ω|1/2nSahKT,eqβexp⁡(−παT,i2ωh3(kT)2|1ln⁡Ω|)     (13)
 MathType@MTEF@5@5@+=feaafiart1ev1aaatCvAUfKttLearuWrP9MDH5MBPbIqV92AaeXatLxBI9gBaebbnrfifHhDYfgasaacH8akY=wiFfYdH8Gipec8Eeeu0xXdbba9frFj0=OqFfea0dXdd9vqai=hGuQ8kuc9pgc9s8qqaq=dirpe0xb9q8qiLsFr0=vr0=vr0dc8meaabaqaciaacaGaaeqabaqabeGadaaakeaacqWGkbGscqGH9aqpdaabdaqaamaalaaabaGaeGymaedabaGagiiBaWMaeiOBa4MaeuyQdCfaaaGaay5bSlaawIa7amaaCaaaleqabaGaeGymaeJaei4la8IaeGOmaidaaOGaemOBa42aaSbaaSqaaiabdofatbqabaGccqWGHbqyieaacqWFObaAcqWGlbWsdaWgaaWcbaGaemivaqLaeiilaWIaemyzauMaemyCaehabeaaiiGakiab+j7aIjGbcwgaLjabcIha4jabcchaWnaabmaabaGaeyOeI0YaaSaaaeaacqGFapaCcqGFXoqydaqhaaWcbaGae8hvaqLaeiilaWIae8xAaKgabaGaeGOmaidaaOGae4xYdCNaemiAaGgabaGaeG4mamJaeiikaGccbeGae03AaSgcbmGaeWhvaqLaeiykaKYaaWbaaSqabeaacqaIYaGmaaaaaOWaaqWaaeaadaWcaaqaaiabigdaXaqaaiGbcYgaSjabc6gaUjabfM6axbaaaiaawEa7caGLiWoaaiaawIcacaGLPaaacaWLjaGaaCzcamaabmaabaGaeGymaeJaeG4mamdacaGLOaGaayzkaaaaaa@6CC8@

where *a *is the lattice spacing and *n*_*s *_is the nucleation site density.

We fit our data from 125 to 160°C to an expanded form of equation 12 (after substitution of equation 13) to describe diopside dissolution as a function of temperature as well as solution composition [7]:

Ri=|ln⁡Ω|16(Ω−1)23(hβiKT,eq(hω2nS,ia)1/3)exp⁡(−Eb,ikT)exp⁡(−παT,i2ωh3(kT)2|1ln⁡Ω|)     (14)
 MathType@MTEF@5@5@+=feaafiart1ev1aaatCvAUfKttLearuWrP9MDH5MBPbIqV92AaeXatLxBI9gBaebbnrfifHhDYfgasaacH8akY=wiFfYdH8Gipec8Eeeu0xXdbba9frFj0=OqFfea0dXdd9vqai=hGuQ8kuc9pgc9s8qqaq=dirpe0xb9q8qiLsFr0=vr0=vr0dc8meaabaqaciaacaGaaeqabaqabeGadaaakeaaieaacqWFsbGudaWgaaWcbaGae8xAaKgabeaakiabg2da9iabcYha8jGbcYgaSjabc6gaUjabfM6axjabcYha8naaCaaaleqabaWaaSGaaeaacqaIXaqmaeaacqaI2aGnaaaaaOGaeiikaGIaeuyQdCLaeyOeI0IaeGymaeJaeiykaKYaaWbaaSqabeaadaWccaqaaiabikdaYaqaaiabiodaZaaaaaGcdaqadaqaaiab=HgaOHGaciab+j7aInaaBaaaleaacqWFPbqAaeqaaOGaem4saS0aaSbaaSqaaiabdsfaujabcYcaSiabdwgaLjabdghaXbqabaGcdaqadaqaaiabdIgaOjab+L8a3naaCaaaleqabaGaeGOmaidaaOGaemOBa42aaSbaaSqaaiabdofatjabcYcaSiabdMgaPbqabaGccqWGHbqyaiaawIcacaGLPaaadaahaaWcbeqaaiabigdaXiabc+caViabiodaZaaaaOGaayjkaiaawMcaaiGbcwgaLjabcIha4jabcchaWnaabmaabaWaaSaaaeaacqGHsislcqWFfbqrdaWgaaWcbaGae8NyaiMaeiilaWIae8xAaKgabeaaaOqaaGqabiab9TgaRjab=rfaubaaaiaawIcacaGLPaaacyGGLbqzcqGG4baEcqGGWbaCdaqadaqaaiabgkHiTmaalaaabaGae4hWdaNae4xSde2aa0baaSqaaiab=rfaujabcYcaSiab=LgaPbqaaiabikdaYaaakiab+L8a3jabdIgaObqaaiabiodaZiabcIcaOiab9TgaRjab=rfaujabcMcaPmaaCaaaleqabaGaeGOmaidaaaaakmaaemaabaWaaSaaaeaacqaIXaqmaeaacyGGSbaBcqGGUbGBcqqHPoWvaaaacaGLhWUaayjcSdaacaGLOaGaayzkaaGaaCzcaiaaxMaadaqadaqaaiabigdaXiabisda0aGaayjkaiaawMcaaaaa@8FE1@

where *i *indicates dissolution due to homogeneous or defect-assisted nucleation of pits on the surface. For ease of discussion, we simplify equation 14 to

Ri=cbiexp⁡(−Eb,ikT)KT,eqexp⁡(παT,i2ωh3(kT)2|1ln⁡Ω|)
 MathType@MTEF@5@5@+=feaafiart1ev1aaatCvAUfKttLearuWrP9MDH5MBPbIqV92AaeXatLxBI9gBaebbnrfifHhDYfgasaacH8akY=wiFfYdH8Gipec8Eeeu0xXdbba9frFj0=OqFfea0dXdd9vqai=hGuQ8kuc9pgc9s8qqaq=dirpe0xb9q8qiLsFr0=vr0=vr0dc8meaabaqaciaacaGaaeqabaqabeGadaaakeaaieaacqWFsbGudaWgaaWcbaGae8xAaKgabeaakiabg2da9iab=ngaJjab=jgaInaaBaaaleaacqWFPbqAaeqaaOGagiyzauMaeiiEaGNaeiiCaa3aaeWaaeaadaWcaaqaaiabgkHiTiab=veafnaaBaaaleaacqWFIbGycqGGSaalcqWFPbqAaeqaaaGcbaGae83AaSMae8hvaqfaaaGaayjkaiaawMcaaiab=TealnaaBaaaleaacqWFubavcqGGSaalcqWFLbqzcqWFXbqCaeqaaOGagiyzauMaeiiEaGNaeiiCaa3aaeWaaeaadaWcaaqaaGGaciab+b8aWjab+f7aHnaaDaaaleaacqWFubavcqGGSaalcqWFPbqAaeaacqaIYaGmaaGccqGFjpWDcqWGObaAaeaacqaIZaWmcqGGOaakieqacqqFRbWAcqWFubavcqGGPaqkdaahaaWcbeqaaiabikdaYaaaaaGcdaabdaqaamaalaaabaGaeGymaedabaGagiiBaWMaeiOBa4MaeuyQdCfaaaGaay5bSlaawIa7aaGaayjkaiaawMcaaaaa@6858@     (15)

where

c=|ln⁡Ω|16(Ω−1)23     (16)
 MathType@MTEF@5@5@+=feaafiart1ev1aaatCvAUfKttLearuWrP9MDH5MBPbIqV92AaeXatLxBI9gBaebbnrfifHhDYfgasaacH8akY=wiFfYdH8Gipec8Eeeu0xXdbba9frFj0=OqFfea0dXdd9vqai=hGuQ8kuc9pgc9s8qqaq=dirpe0xb9q8qiLsFr0=vr0=vr0dc8meaabaqaciaacaGaaeqabaqabeGadaaakeaacqWGJbWycqGH9aqpdaabdaqaaiGbcYgaSjabc6gaUjabfM6axbGaay5bSlaawIa7amaaCaaaleqabaWaaSGaaeaacqaIXaqmaeaacqaI2aGnaaaaaOGaeiikaGIaeuyQdCLaeyOeI0IaeGymaeJaeiykaKYaaWbaaSqabeaadaWccaqaaiabikdaYaqaaiabiodaZaaaaaGccaWLjaGaaCzcamaabmaabaGaeGymaeJaeGOnaydacaGLOaGaayzkaaaaaa@44AD@

and

*b*_*i *_= *hβ*_*i*_(*hω*^2^*n*_*s*, *i*_*a*)^1/3 ^    (17)

The total dissolution rate is simply the summation of dissolution due to both mechanisms:

*R*_*net *_= *R*_*homogeneous *_+ *R*_*defect-assisted *_    (18)

At a fixed temperature, *b*_*i *_and α_*i *_can be derived from a linear form of equation 15 by normalizing *R*_*i *_to solution saturation (*c *defined by equation 16) and applying the natural log:

ln⁡(Ric)=ln⁡(bT,iKT,eq)−παT,i2ωh3(kT)2|1ln⁡Ω|     (19)
 MathType@MTEF@5@5@+=feaafiart1ev1aaatCvAUfKttLearuWrP9MDH5MBPbIqV92AaeXatLxBI9gBaebbnrfifHhDYfgasaacH8akY=wiFfYdH8Gipec8Eeeu0xXdbba9frFj0=OqFfea0dXdd9vqai=hGuQ8kuc9pgc9s8qqaq=dirpe0xb9q8qiLsFr0=vr0=vr0dc8meaabaqaciaacaGaaeqabaqabeGadaaakeaacyGGSbaBcqGGUbGBdaqadaqaamaalaaabaacbaGae8Nuai1aaSbaaSqaaiab=LgaPbqabaaakeaacqWFJbWyaaaacaGLOaGaayzkaaGaeyypa0JagiiBaWMaeiOBa42aaeWaaeaacqWFIbGydaWgaaWcbaGae8hvaqLaeiilaWIae8xAaKgabeaakiabdUealnaaBaaaleaacqWGubavcqGGSaalcqWGLbqzcqWGXbqCaeqaaaGccaGLOaGaayzkaaGaeyOeI0YaaSaaaeaaiiGacqGFapaCcqGFXoqydaqhaaWcbaGae8hvaqLaeiilaWIae8xAaKgabaGaeGOmaidaaOGae4xYdCNaemiAaGgabaGaeG4mamJaeiikaGccbeGae03AaSMae8hvaqLaeiykaKYaaWbaaSqabeaacqaIYaGmaaaaaOWaaqWaaeaadaWcaaqaaiabigdaXaqaaiGbcYgaSjabc6gaUjabfM6axbaaaiaawEa7caGLiWoacaWLjaGaaCzcamaabmaabaGaeGymaeJaeGyoaKdacaGLOaGaayzkaaaaaa@6509@

Changes in mineral dissolution as a function of temperature are accounted for by *β*_*i *_and *n*_*s*,*i *_in the y-intercept and in α_*i *_in the slope in addition to the saturation terms (Ω, K_T,eq_) and T in equation 19. The temperature dependence of *β*_*i *_and *n*_*s*,*i *_can be estimated collectively from the Arrhenius equation:

∂ln⁡b∂1T=Ebk,     (20)
 MathType@MTEF@5@5@+=feaafiart1ev1aaatCvAUfKttLearuWrP9MDH5MBPbIqV92AaeXatLxBI9gBaebbnrfifHhDYfgasaacH8akY=wiFfYdH8Gipec8Eeeu0xXdbba9frFj0=OqFfea0dXdd9vqai=hGuQ8kuc9pgc9s8qqaq=dirpe0xb9q8qiLsFr0=vr0=vr0dc8meaabaqaciaacaGaaeqabaqabeGadaaakeaadaWcaaqaaGGaciab=jGi2kGbcYgaSjabc6gaUHqaaiab+jgaIbqaaiab=jGi2oaalaaabaGaeGymaedabaGae4hvaqfaaaaacqGH9aqpdaWcaaqaaiab+veafnaaBaaaleaacqGFIbGyaeqaaaGcbaacbeGae03AaSgaaiabcYcaSiaaxMaacaWLjaWaaeWaaeaacqaIYaGmcqaIWaamaiaawIcacaGLPaaaaaa@405B@

where *E*_*b *_is the kinetic barrier. It is not possible to resolve the temperature dependence of *β*_*i *_and *n*_*s*,*i *_separately with our data set. The temperature dependence of α_*i *_can be estimated from a variation of the Gibbs-Hemholtz equation:

∂α∂1T=ΔH,     (21)
 MathType@MTEF@5@5@+=feaafiart1ev1aaatCvAUfKttLearuWrP9MDH5MBPbIqV92AaeXatLxBI9gBaebbnrfifHhDYfgasaacH8akY=wiFfYdH8Gipec8Eeeu0xXdbba9frFj0=OqFfea0dXdd9vqai=hGuQ8kuc9pgc9s8qqaq=dirpe0xb9q8qiLsFr0=vr0=vr0dc8meaabaqaciaacaGaaeqabaqabeGadaaakeaadaWcaaqaaGGaciab=jGi2kab=f7aHbqaaiab=jGi2oaalaaabaGaeGymaedabaGaemivaqfaaaaacqGH9aqpcqGHuoarcqWGibascqGGSaalcaWLjaGaaCzcamaabmaabaGaeGOmaiJaeGymaedacaGLOaGaayzkaaaaaa@3C66@

where ΔH is the enthalpy associated with the step edge energy for pit nucleation.

**Figure 9:**
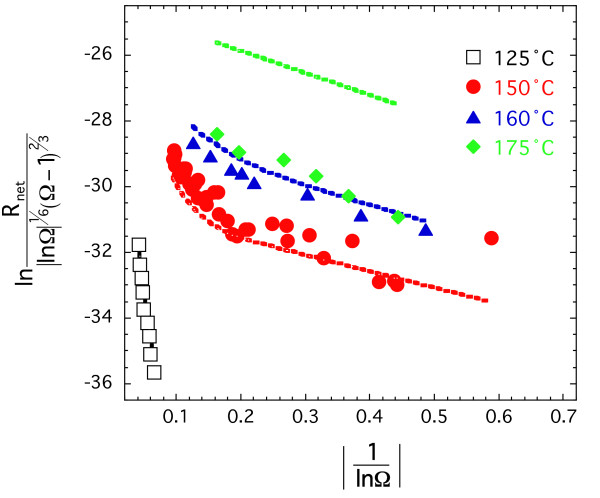
Natural log of normalized dissolutions rates obtained at different temperatures are plotted against |1ln⁡Ω|
 MathType@MTEF@5@5@+=feaafiart1ev1aaatCvAUfKttLearuWrP9MDH5MBPbIqV92AaeXatLxBI9gBaebbnrfifHhDYfgasaacH8akY=wiFfYdH8Gipec8Eeeu0xXdbba9frFj0=OqFfea0dXdd9vqai=hGuQ8kuc9pgc9s8qqaq=dirpe0xb9q8qiLsFr0=vr0=vr0dc8meaabaqaciaacaGaaeqabaqabeGadaaakeaadaabdaqaamaalaaabaGaeGymaedabaGagiiBaWMaeiOBa4MaeuyQdCfaaaGaay5bSlaawIa7aaaa@3522@. Best fits with a pit nucleation model to the experimental data are shown by similar color lines (see text for detail).

**Figure 10:**
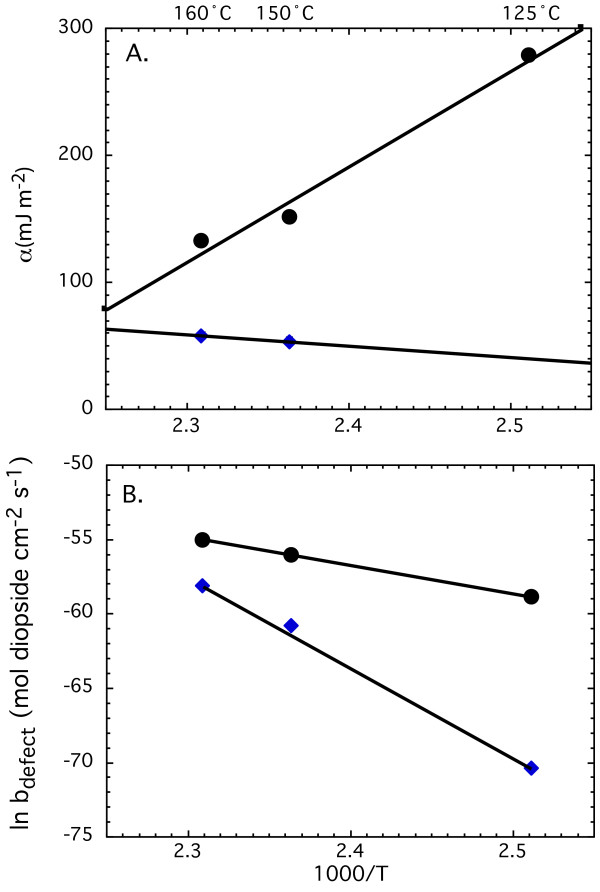
Temperature dependence of pit nucleation model parameters: (A) step edge energy and (B) ln *b *for homogenous nucleation of pits is shown by black circles and those for defect-assisted pits are shown by blue triangles. The lines represent the best fits to the data.

Final fits to the data are shown in Figure 9 and 10 and Table 3. To fit the data as a function of temperature, we first fit data sets at each temperature assuming that data collected at 125°C resulted from dissolution promoted by homogeneous nucleation of pits and that data collected at 150 and 160°C resulted from dissolution promoted by homogenous nucleation of pits and defect-assisted nucleation of pits. The initial allocation of mechanism was based on the shape of the curve and its location in saturation space, where higher degrees of undersaturation (i.e. small 1/lnΩ and steeper slopes) are likely to result in the homogeneous nucleation of dissolution pits and where solutions closer to equilibrium (i.e. larger 1/lnΩ and flatter slopes) are likely to result in defect-assisted nucleation of pits. Temperature dependence was then evaluated using equations 20 and 21 (Figure 10) and extrapolated to 175°C, because the highly linear and limited data set at this temperature did not allow contributions of homogeneous and defect-assisted nucleation to be constrained.

**Table 3:** Pit Nucleation Model. Fitted parameters for equations 20 and 21 needed to describe diopside dissolution as a function of temperature.

ΔH_α-homogeneous _= 749,700 mJ m^-2^					
ΔH_α-defect assisted _= -91,644 mJ m^-2^					
E_b-homogeneous _= 2.59 × 10^-16 ^mJ K^-1^, ln b_homogeneous _= -11.57 mol cm^-2 ^s^-1^					
E_b-defect assisted _= 8.44 × 10^-16 ^mJ K^-1^, ln b_defect assisted _= 83.34 mol cm^-2 ^s^-1^					
ω = 1.1 × 10^-28 ^m^3^, h = 5.25 × 10^-10 ^m					
**T°C**	**^1^K_eq_**	**α_homogenous _**mJ m^-2^	**^2^y-intercept_homogeneous_**	**α_defect assited _**mJ m^-2^	**^2^y-intercept_defect-assisted_**

125	10^14.48^	275.9	-25.5	39.4	-36.9
150	10^13.27^	164.6	-25.5	53.0	-30.6
160	10^12.82^	123.7	-25.5	58	-28.3
175	10^12.19^	^3^65.8	^3^-25.5	^3^65.1	^3^-25.0

^1^Solubility constants K_eq _are taken from Supcrit92 (Johnson et al., 1992).

^2^Calculated from: y-intercept_T,i _= ln K_T,eq _+ ln b_i _- E_b T,i_/kT

^3^Values for 175°C were extrapolated based on temperature dependence of best fit values at 125, 150, and 160°C.

Final fits to the data indicate that dissolution is promoted predominately by homogenous nucleation at 125°C over the narrow range of solution saturation (1/lnΩ < 0.07) studied here. At 150 and 160°C dissolution is promoted by both homogeneous and defect-assisted nucleation of pits such that homogeneous nucleation is negligible at 1/lnΩ > 0.25 where it contributes less than 2% to the total dissolution rate. Extrapolation of the model to 175°C indicates that steady-state dissolution rates can be attributed to homogeneous and defect-assisted nucleation mechanisms in roughly equal proportions over the limited saturation range in this study. There is significant mismatch between the model prediction and diopside dissolution at 175°C. The most likely explanation for the mismatch is that the measured rates represent both dissolution of diopside and the precipitation of a secondary phase. Mineral precipitation was also indicated with the ion exchange model (see section 4).

Our results show that step edge energy for homogeneous nucleation is generally higher than step edge energy for defect-assisted nucleation, consistent with the observations for quartz, feldspar, and kaolinite [7]. However the difference between α_homogenous _and α_defect-assited _decreases at higher temperature, because estimated step edge energies for homogeneous and defect-assisted nucleation have different temperature dependencies. A decrease in step edge energy for homogeneous nucleation of pits at the diopside surface from about 275 to 65 mJ m^-2 ^from 125 to 175°C suggests that the step edge energy required to form pits on an otherwise perfect crystal surface is lower at higher temperatures. There appears to be little dependence of the homogeneous pit site density or the kinetic coefficient on temperature as is illustrated by near constant y-intercept for the contribution of homogeneous nucleation of pits to diopside dissolution (Table 3). In contrast to homogeneous nucleation of dissolution pits, the temperature dependence of defect-assisted nucleation of dissolution pits on the diopside surface increases slightly with increasing temperature from about 39 to 65 mJ m^-2 ^from 125 to 175°C. This increase suggests that defect-assisted pits form more readily at lower temperature than at higher temperature. Ostensibly higher step edge energy for defect-assisted nucleation at higher temperature appears to be compensated by an increase in the combined kinetic coefficient and site density for defect-assisted nucleation. Thus as the step edge energy rises with temperature, the kinetic barrier is lowered by increasing the number of defects that are accessible at higher temperature. The net result is higher dissolution rates at higher temperature at conditions closer to equilibrium where defect-assisted nucleation of dissolution pits are expected to dominate.

## 6. Broad implications for developing predictive geochemical models

**Figure 11:**
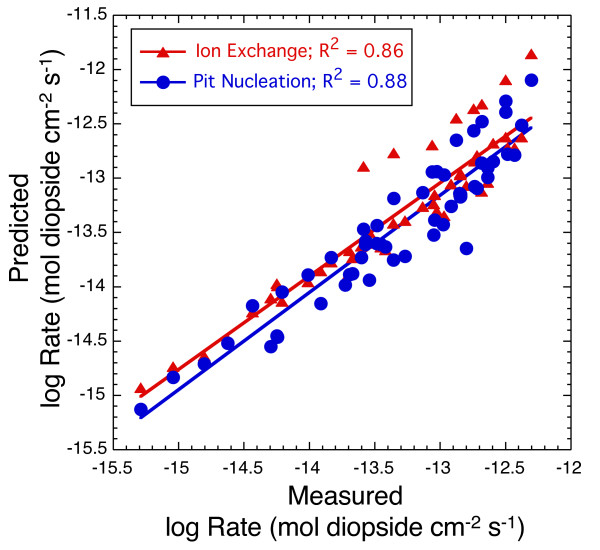
Comparison of the predicted log rates (diopside cm^-2 ^s^-1^) using the ion exchange and pit nucleation models with the measured log rates at 125, 150 and 160°C. Comparison of predicted and measured rates at 175°C was not made because both models significantly over predict dissolution compared to the measured rates.

Diopside dissolution can be described equally well by both an ion exchange model based on transition state theory and a pit nucleation model based on crystal growth/dissolution theory from 125 to 160°C (Figure 11), and both models predict much higher dissolution rates at 175°C than those measured indicating secondary mineral precipitation in the experiments. Thus based on the fitted data, we cannot determine if diopside kinetics are controlled by reversible reactions at the mineral surface (transition state theory) or if they are controlled by combined homogeneous and defect-assisted nucleation of pits on the mineral surface (crystal growth/dissolution theory). It was not possible to isolate pits due to homogeneous nucleation and defect-assisted nucleation by imagining gem stone quality diopside surfaces reacted at 150°C at distinct saturations representative of the two mechanism, as was done for quartz [7], because similar dissolution features and surface roughness were observed in both regions (interferometry data not shown). It is not clear if dissolution features were artifacts of the gem polishing technique or represented combined contributions from homogeneous and defect-assisted nucleations pits as predicted by fitted results of the macroscopic data.

Both these dissolution models are based on sound thermodynamic and kinetic principles, however, the mechanism on which they are based on are very different. Both models link kinetic rates to solution composition through the Gibbs free energy of reaction or solution saturation, and they are a significant improvement on the use of rate constants derived at conditions far from equilibrium and the principle of detailed balancing to describe rock-water processes important to soil formation, weathering, diagenesis, and environmental issues such as radioactive waste disposal and CO_2 _sequestration. However successful application of models as a predictive tool requires that they be experimentally calibrated. Here we briefly discuss calibration experiments needed to develop each of these models for a given mineral system.

Calibration of the ion exchange model requires that mineral dissolution rates be measured over a range of solution saturation and temperature at a single pH (at a minimum). The precursor forming exchange reactants (i.e. Mg-H for magnesio-silicates and Al-H for alumino-silicates) can be predicted from the relative dissolution rates of single hydroxides [15] and is related to the leached layer composition of the dissolving mineral. The exchange co-efficient (*n *in equation 6) is the number of cations removed to form the precursor complex should be determined empirically. Previous studies on alumino-silicate minerals suggested that *n *can be predicted from the charge balance where three protons are exchanged for each alumina [18]. This was not the case for diopside and may not be the case for other minerals. The apparent rate constant (*k *in equation 8) must also be determined empirically as a function of temperature to derive the apparent activation energy. Ideally, the effect of pH can be determined from experiments conducted at a single value, because pH is accounted for in the exchange reaction to form the Si-rich precursor (as shown in equation 6 for diopside). For enstatite dissolution, a model constrained at pH 2 is able to describe dissolution rates from pH 2 to 10 [17]. Similarly, for basaltic glass dissolution, the same model parameters describe dissolution at pH 3 and 11 [13]. In contrast, model parameters obtained at acid pH for kaolinite and muscovite dissolution are different from those obtained at basic pH conditions [11,15].

Compared to the ion exchange model based on transition state theory, much more experimental data are required for the development and validation of a model based on crystal growth/dissolution theory. Mineral dissolution rates based on crystal growth/dissolution theory are dependent on the dominant source of steps. The source of steps can be at existing dislocations, existing crystal edges, nucleated homogeneously throughout the mineral surface or nucleated at specific defect sites. In the absence of experimental data (either microscopic or macroscopic), the source of steps cannot be determined *a priori *and are dependent on temperature and the extent of saturation for a given source of steps. For example Dove et al [7] showed that kaolinite dissolution rates obtained at 80°C are best explained by retreat of steps originating at dislocations. In contrast, rates obtained at 150°C are best explained by the pit nucleation model. The effect of solution pH is explicitly accounted for in the saturation terms and has been validated for kaolinite dissolution data obtained at 150°C under acid and circum-neutral pH conditions. However, the solution saturation ranges for homogeneous and defect-assisted nucleation of pits cannot be determined *a priori*. Even when the dominant step type is determined from microscopic observations, experimental dissolution data obtained over a range of saturation and temperature are still needed to empirically derive the temperature dependence for the step edge energy, site density, and kinetic coefficient.

**Figure 12:**
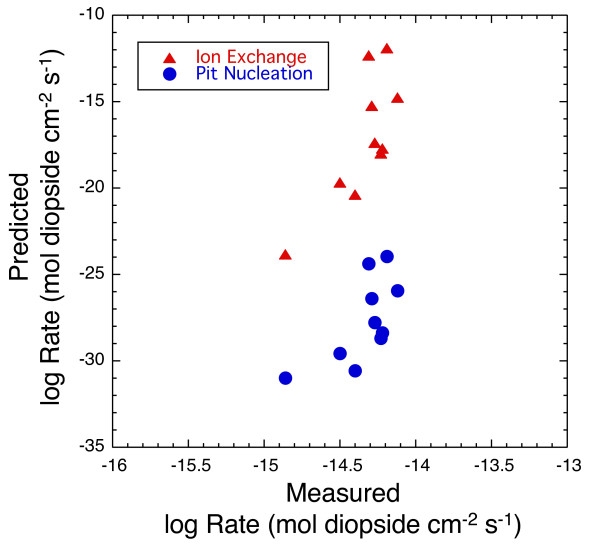
Extrapolation of the ion exchange and pit nucleation models to 25°C using parameters calibrated with the data in this study between 125 and 160°C (Equations 9 and 14). Measured diopside rate data and solution compositions are from Golubev et al. [35]. All rates are given as log rates (mol diopside cm^-2 ^s^-1^).

Caution should be applied when extending dissolution models outside of their calibration range. Figure 12 compares diopside dissolution rates calculated from the ion exchange and pit nucleation models using parameters calibrated with the data in this study between 125 and 160°C (Equations 9 and 14) with measured diopside dissolution rates at 25°C. Measured diopside rate data and solution compositions are from Golubev et al. [35]; solution speciation and were calculated using Supcrit92 thermodynamic data base where log K_eq _= 20.96 for diopside solubility [22]. Comparisons between predicted and measured rates are made only for those experiments with reported pH and dissolved Mg, Ca, and Si concentrations. Rates are based on the stoichiometric release of Si, pH ranged from 1 to 5.05, and solutions were highly undersaturated with respect to diopside equilibrium, ΔGr < -130 (kJ mol^-1^). Both models calibrated with the high temperature data under predict measured rates at 25°C. For the ion exchange model, the large discrepancy suggests lower activation energy at lower temperature consistent with experimental studies [29-32] and/or pH dependent parameters as is the case for Al-silicates [11,15]. For the pit nucleation model, the large discrepancy may indicate that step retreat controls diopside dissolution at 25°C as has been proposed for kaolinite at 80°C [7], or that activation energy and enthalpy terms associated with step edge energy, site density, and kinetic coefficients are different at lower temperature.

A final note is that the precipitation rate expressions are needed to fully describe many rock-water interactions in the near surface. This is clearly illustrated in our experiments where mineral precipitation is indicated by similar rates measured at 160 and 175°C and by the mismatch between model predictions and measured rates.
